# Efficacy of silver modified atraumatic restorative treatment (SMART) versus atraumatic restorative treatment (ART) for caries control in primary teeth: a systematic review and meta-analysis

**DOI:** 10.3389/fdmed.2026.1914881

**Published:** 2026-08-19

**Authors:** Katia Noemi Rubio-Membrillo, María Victoria Espinoza-Salcedo, Heber Isac Arbildo-Vega, Omar Edmundo Rojas-Rojas, Edith Esther Delgado-Asmat, Jhair Alexander León-Rodríguez, Franz Tito Coronel-Zubiate

**Affiliations:** 1 Graduate School, Universidad Privada Antenor Orrego, Trujillo, Peru; 2 Faculty of Dentistry, Dentistry School, Universidad San Martín de Porres, Chiclayo, Peru; 3 Faculty of Human Medicine, Human Medicine School, Universidad San Martín de Porres, Chiclayo, Peru; 4 Graduate School, Universidad Nacional Toribio Rodríguez de Mendoza de Amazonas, Chachapoyas, Peru; 5 Faculty of Health Sciences, Stomatology School, Universidad Nacional Toribio Rodríguez de Mendoza de Amazonas, Chachapoyas, Peru

**Keywords:** atraumatic restorative treatment, dental caries, meta-analysis, primary teeth, silver diamine fluoride, systematic review

## Abstract

**Background:**

Early childhood caries represents a severe global public health problem. Faced with this, Atraumatic Restorative Treatment (ART) and Silver Modified Atraumatic Restorative Treatment (SMART) have positioned themselves as minimally invasive approaches. The objective of this study was to evaluate and compare the clinical efficacy of SMART versus conventional ART for caries control in primary teeth.

**Methods:**

A systematic review and meta-analysis were conducted in accordance with the PRISMA guidelines, searching for randomized clinical trials (RCTs) across seven databases up to March 2026. Studies with a minimum follow-up period of 6 months were included. The risk of bias was assessed using the RoB 2 tool, and the certainty of evidence was evaluated using the GRADE approach.

**Results:**

Seven reports derived from six randomized clinical trials with an overall low risk of bias were included in the qualitative synthesis, representing 398 unique pediatric participants. One trial generated two complementary publications reporting different outcomes; therefore, only the publication reporting clinical success contributed to the quantitative synthesis. A total of six randomized clinical trials were included in the meta-analysis, which showed no statistically significant differences in overall clinical success between SMART and ART (OR = 1.28; 95% CI: 0.91–1.80; *p* = 0.16). Furthermore, Trial Sequential Analysis (TSA) demonstrated that the cumulative evidence remains inconclusive.

**Conclusions:**

There is no statistically significant difference in overall clinical success between SMART and ART for the primary dentition. Both are viable and effective strategies; however, the certainty of the available evidence is low, and therefore future large-scale RCTs are required to establish definitive clinical guidelines.

**Systematic Review Registration:**

https://www.crd.york.ac.uk/PROSPERO/view/CRD420261325936, PROSPERO CRD420261325936.

## 1 Introduction

Early childhood caries represents a persistent global public health crisis affecting a vast proportion of preschoolers, overwhelming current healthcare systems (1). This infectious and chronic disease generates a devastating clinical impact, causing acute pain, nutritional difficulties, and severe alterations in the long-term cognitive and maxillofacial development of the pediatric patient (2–4). Despite international preventive efforts, traditional strategies have systematically failed in diverse populations due to multiple socioeconomic barriers and lack of access to clinical infrastructure, shaping a high morbidity burden from untreated carious lesions (1, 5).

Faced with this adverse epidemiological panorama, ART emerged as one of the pioneering minimally invasive restorative approaches for managing dental caries in community settings with limited resources (6–8). Its philosophy subsequently became one of the fundamental pillars supporting the broader concept of minimally invasive dentistry, emphasizing maximum preservation of tooth structure while providing effective disease control. This approach eliminates the need for noisy rotary equipment and local infiltrative anesthesia, significantly reducing dental anxiety and improving the behavioral cooperation of the pediatric patient (9–11). Thus, the procedure facilitates the provision of therapeutic care in vulnerable areas, seeking to arrest disease progression by altering the bacterial microenvironment without requiring complex surgical instruments (12).

The standardized ART protocol bases its biomechanical success on the selective manual removal of infected superficial dentin, preserving intact the deep dentin which possesses high remineralization potential (7, 10). Subsequently, the cavitated defect is hermetically sealed using high-viscosity glass ionomer cements, selected for their excellent intrinsic adhesive properties and proven pulpal biocompatibility (13). The gradual, constant, and sustained release of fluoride ions by this restorative material contributes to effectively halting the metabolic progression of underlying residual lesions, ensuring tissue arrest (14, 15).

Although ART has demonstrated favorable clinical performance, particularly in single-surface cavitated lesions in primary teeth, restoration longevity tends to decrease in multisurface or occlusoproximal cavities because of increased biomechanical stresses and reduced mechanical retention (16, 17). Consequently, cavity characteristics constitute one of the main determinants of long-term clinical success. The main biomechanical cause of this premature failure lies in deficient micromechanical retention and the rapid structural degradation of the material under continuous masticatory loads (17). Furthermore, the persistence of cariogenic bacterial biomass demands more robust antimicrobial properties, as incomplete manual removal substantially increases the biological risk of secondary lesions, demanding a critical reevaluation of cavity protocols (14).

To overcome these profound biological and retentive limitations, silver diamine fluoride (SDF) has rapidly positioned itself as a synergistic and highly cost-effective cariostatic agent (18). This innovative liquid formulation combines the potent lethal bactericidal effect of silver ions with the high remineralizing capacity of fluoride, inducing the formation of fluorapatite and sclerosing dentinal tubules almost immediately (19, 20). Its professional application arrests active cariogenic activity in a vast majority of treated lesions, radically transforming the care paradigm by allowing fast and safe interventions in pre-cooperative patients or those with complex special needs (21, 22).

The clinical integration of this potent liquid preventive agent with subsequent occlusal sealing gave rise to the revolutionary SMART (18, 23). This cutting-edge hybrid technique provides a dual protection mechanism: it strategically leverages the biological arrest of demineralization while simultaneously restoring anatomical morphology and masticatory function with glass ionomer (24, 25). Theoretically, SDF preconditioning may improve the biological environment of the prepared cavity through its antibacterial activity and dentin-hardening effect, potentially favoring restoration longevity (26–28). Nevertheless, whether these biological effects consistently translate into improved marginal adaptation and long-term adhesive performance remains a matter of ongoing debate.

However, the methodological integration of both compounds generates substantial controversies regarding the integrity of the adhesive interface and clinical stability, with comparative evidence remaining ambiguous and fragmented. Recent laboratory investigations yield discrepant results on whether the metallic precipitate negatively interferes with the long-term micromechanical bond between the modified dentin and the ionomer cement (26, 29). Added to the questions concerning aesthetic acceptability due to the inherent dark pigmentation (30), it is imperative to consolidate these disparate findings to clarify whether the biological advantage truly translates into lower caries recurrence compared to traditional therapeutic options (31–33).

From a clinical perspective, despite their numerous clinical advantages, both ART and SMART should be applied under appropriate clinical conditions. These minimally invasive approaches are primarily indicated for cavitated dentin lesions without signs of irreversible pulpal involvement, while teeth presenting spontaneous pain, pulpal necrosis, abscess formation, or extensive structural destruction generally require alternative therapeutic strategies. Likewise, clinical success depends on adequate case selection and the possibility of completing the restorative procedure under acceptable behavioral conditions.

Addressing this methodological uncertainty is crucial to establish solid evidence-based clinical guidelines that optimize the comprehensive management of this childhood disease. Rigorously synthesizing randomized controlled trials will allow health professionals to adequately weigh the prophylactic benefits against the possible biomechanical disadvantages of the modified technique. Despite previous narrative reviews and individual clinical studies, high-quality evidence directly comparing SMART and conventional ART remains limited and fragmented, warranting an updated systematic review and meta-analysis focused on randomized controlled trials. Therefore, the main objective of this systematic review and meta-analysis is to evaluate and compare the clinical efficacy of SMART versus conventional ART for caries control in the primary dentition.

## 2 Article types

Systematic review.

## 3 Materials and methods

### 3.1 Protocol and registration

This systematic review was conducted in accordance with the Preferred Reporting Items for Systematic Reviews and Meta-Analyses (PRISMA) guidelines (34). The protocol was registered in the International Prospective Register of Systematic Reviews (PROSPERO) (35) under the registration number CRD420261325936. The completed PRISMA 2020 Checklist is available as Supplementary Material 1.

### 3.2 Definitions of interventions

- Atraumatic Restorative Treatment (ART): This intervention involves the selective removal of infected superficial dentin using exclusively hand instruments, eliminating the need for rotary equipment or local infiltrative anesthesia. The prepared cavity and adjacent fissures are subsequently restored and hermetically sealed using high-viscosity glass ionomer cement (HVGIC).
- Silver Modified Atraumatic Restorative Treatment (SMART): This hybrid intervention consists of the topical application of silver diamine fluoride (SDF) to the active carious lesion as a chemical preconditioning and cariostatic agent, followed by the restoration and occlusal sealing of the defect with HVGIC. Like ART, this procedure is performed without the use of rotary instruments or local anesthesia.
- High-Viscosity Glass Ionomer Cement (HVGIC): These represent the standard restorative biomaterials utilized in both the experimental and control groups of the included studies. They are selected for their intrinsic chemical adhesion to tooth structure, pulpal biocompatibility, and sustained fluoride-releasing properties.
- Silver Diamine Fluoride (SDF): A topical liquid formulation utilized exclusively in the experimental cohorts (SMART), combining the bactericidal effect of silver ions with the remineralizing capacity of fluoride to arrest active cariogenic progression prior to the placement of the final restoration.

### 3.3 Focused question and PICO framework

The focused question was formulated using the PICO format (population, intervention, comparison and outcomes), as detailed below:

- Population: Pediatric patients with cavitated carious lesions in primary teeth.
- Intervention: Silver Modified Atraumatic Restorative Treatment (SMART).
- Comparison: Atraumatic Restorative Treatment (ART).
- Outcomes: Clinical success, radiographic success, arrested caries, pain, quality of life, aesthetic perception, and total cost.

Focused question (PICO): Is Silver Modified SMART more effective than conventional ART for controlling cavitated carious lesions in pediatric patients?

### 3.4 Information sources and search strategy

For this systematic review, a comprehensive search was performed in seven electronic databases (PubMed, Cochrane Library, Scopus, Web of Science, Embase, LILACS, and SciELO) and two grey literature sources (Google Scholar and ProQuest) up to March 2026. The search strategy utilized a combination of database-specific controlled vocabulary and keywords such as “Dental Caries”, “primary teeth”, “Atraumatic Restorative Treatment”, and “SMART” (full search strategies are detailed in Table 1). Furthermore, the reference lists of all included studies were hand-searched to identify additional relevant literature.

**Table 1:** Search and selection of studies.

Database	Search strategy	Number of study
PubMed	(("Tooth, Deciduous"[MeSH Terms] OR "Dental Caries"[MeSH Terms] OR "deciduous tooth"[Title/Abstract] OR "primary teeth"[Title/Abstract] OR "primary dentition"[Title/Abstract] OR "milk teeth"[Title/Abstract] OR "baby teeth"[Title/Abstract] OR "pediatric*"[Title/Abstract] OR "paediatric*"[Title/Abstract] OR "child*"[Title/Abstract] OR "caries"[Title/Abstract] OR "carious"[Title/Abstract]) AND ("Atraumatic Restorative Treatment"[Title/Abstract] OR "ART"[Title/Abstract]) AND ("SMART"[Title/Abstract] OR "Silver Modified Atraumatic Restorative Treatment"[Title/Abstract] OR "Silver Diamine Fluoride"[Title/Abstract] OR "SDF"[Title/Abstract] OR "Silver Nitrate"[Title/Abstract] OR "Glass Ionomer*"[Title/Abstract]) AND (randomized controlled trial[pt] OR controlled clinical trial[pt] OR randomized[tiab] OR placebo[tiab] OR "clinical trials as topic"[mesh:noexp] OR randomly[tiab] OR trial[tiab] OR groups[tiab]))	203
Cochrane Library	([mh "Tooth, Deciduous"] OR [mh "Dental Caries"] OR "primary teeth":ti,ab,kw OR "primary dentition":ti,ab,kw OR "deciduous tooth":ti,ab,kw OR "caries":ti,ab,kw) AND ([mh "Dental Atraumatic Restorative Treatment" OR "ART":ti,ab,kw] AND ["SMART":ti,ab,kw OR "SDF":ti,ab,kw OR "Silver Modified Atraumatic Restorative Treatment":ti,ab,kw)	70
Scopus	(TITLE-ABS-KEY (("deciduous tooth" OR "primary teeth" OR "primary dentition" OR "child*" OR "caries") AND TITLE-ABS-KEY ("Atraumatic Restorative Treatment" OR "ART") AND TITLE-ABS-KEY ("SMART" OR "Silver Diamine Fluoride" OR "SDF") AND TITLE-ABS-KEY ("randomized" OR "trial" OR "controlled")))	71
Web of Science	("deciduous tooth" OR "primary teeth" OR "primary dentition" OR "caries") (Topic) AND ("Atraumatic Restorative Treatment" OR "ART") (Topic) AND ("SMART" OR "Silver Diamine Fluoride" OR "SDF") (Topic) AND ("random*" OR "trial") (Topic)	55
Embase	(‘deciduous tooth'/exp OR ‘dental caries’/exp OR ‘primary teeth':ab,ti OR ‘primary dentition':ab,ti OR ‘milk teeth':ab,ti OR ‘baby teeth':ab,ti OR ‘child':ab,ti OR ‘pediatric':ab,ti OR ‘paediatric':ab,ti OR ‘caries':ab,ti) AND (‘atraumatic restorative treatment'/exp OR ‘art':ab,ti) AND (’silver diamine fluoride'/exp OR ‘smart':ab,ti OR ‘sdf':ab,ti OR ‘silver modified atraumatic restorative treatment':ab,ti OR ‘glass ionomer':ab,ti) AND (‘randomized controlled trial'/exp OR ‘randomization'/exp OR ‘random*':ab,ti OR ‘trial':ab,ti)	199
LILACS	(tw:("silver modified atraumatic restorative technique" OR "SMART" OR "silver diamine fluoride" OR "diamino fluoruro de plata" OR "diamino fluoreto de prata" OR "SDF")) AND (tw:("atraumatic restorative treatment" OR "ART" OR "tratamiento restaurador atraumático" OR "tratamento restaurador atraumático")) AND (tw:("primary teeth" OR "deciduous teeth" OR "dientes deciduos" OR "dentes decíduos" OR "dientes temporales"))	7
Scielo	(ti:(caries OR "dientes deciduos" OR "dentes decíduos" OR "primary teeth")) AND (ab:(SMART OR "SDF" OR "fluoruro diamino de plata" OR "diamino fluoreto de prata")) AND (ab:(ART OR "tratamiento restaurativo atraumatico" OR "tratamento restaurador atraumático"))	0
Google Scholar	"Silver Modified Atraumatic Restorative Treatment" SMART ART "Silver Diamine Fluoride" caries "primary teeth" trial	108
Proquest	TI,AB("primary teeth" OR "deciduous tooth" OR "child*") AND TI,AB("SMART" OR "Silver Modified Atraumatic Restorative Treatment" OR "Silver Diamine Fluoride") AND TI,AB(random* OR trial) AND TI,AB("Atraumatic") NOT TI,AB("meta-analysis" OR "review")	8

### 3.5 Study selection

Two authors (KR and HA) independently screened the search results and performed the final study selection. Articles were included based on the following criteria: randomized clinical trials (RCTs), a minimum follow-up period of 6 months, and reporting on the efficacy of SMART versus conventional ART for caries control in the primary dentition. No restrictions regarding publication year or publication language were applied. Studies published in languages other than English were eligible for inclusion, provided that sufficient information was available for data extraction and methodological assessment. Non-randomized prospective studies, unpublished reports, and duplicate publications of the same cohort with varying follow-up periods were excluded.

### 3.6 Data extraction

Data extraction from the eligible studies was performed independently by two investigators (OR and FC) using a standardized, pre-piloted form. Any disagreements were resolved through discussion and consensus with a third reviewer (ME). The extracted variables included: author(s), publication year, study design, country of origin, sample size, sex distribution, mean age and age range, follow-up duration, funding source, inclusion and exclusion criteria, outcome measurement instruments, and detailed characteristics of the study groups (including the number of patients and teeth treated per group). Outcome data extracted encompassed clinical and radiographic success, overall caries arrest rates (with specific data for Class I and II lesions), pain, quality of life, aesthetic perception, and total cost.

### 3.7 Risk of bias (RoB) assessment

The risk of bias (RoB) of the included trials was independently assessed by two calibrated reviewers (ED and JL), both with previous experience in conducting systematic reviews and in the application of the revised Cochrane risk-of-bias tool for randomized trials (RoB 2) (36), who achieved excellent inter-rater agreement (*κ* = 0.95). Any discrepancies were resolved by consulting a third reviewer (HA). According to this tool, trials were evaluated across five domains: bias arising from the randomization process, deviations from intended interventions, missing outcome data, measurement of the outcome, and selection of the reported result. The overall risk of bias for each study was subsequently classified as “low risk”, “some concerns”, or “high risk”.

### 3.8 Statistical analysis and certainty of evidence (GRADE)

Statistical analysis was performed using Stata version 17.0 (StataCorp LLC, College Station, TX, USA). For dichotomous outcomes, pooled effect estimates were calculated using the Mantel-Haenszel method. Fixed-effects models were applied when no statistical heterogeneity was detected (*I*^2^ < 50%), whereas random-effects models were planned for analyses showing substantial heterogeneity. Outcome measures were expressed as odds ratios (ORs) with 95% confidence intervals (CIs). Additionally, the certainty of the body of evidence was evaluated using the Grading of Recommendations Assessment, Development and Evaluation (GRADE) approach, utilizing the GRADEpro Guideline Development Tool (GDT) (McMaster University and Evidence Prime Inc., Canada).

## 4 Results

### 4.1 Selection of studies

**Figure 1:**
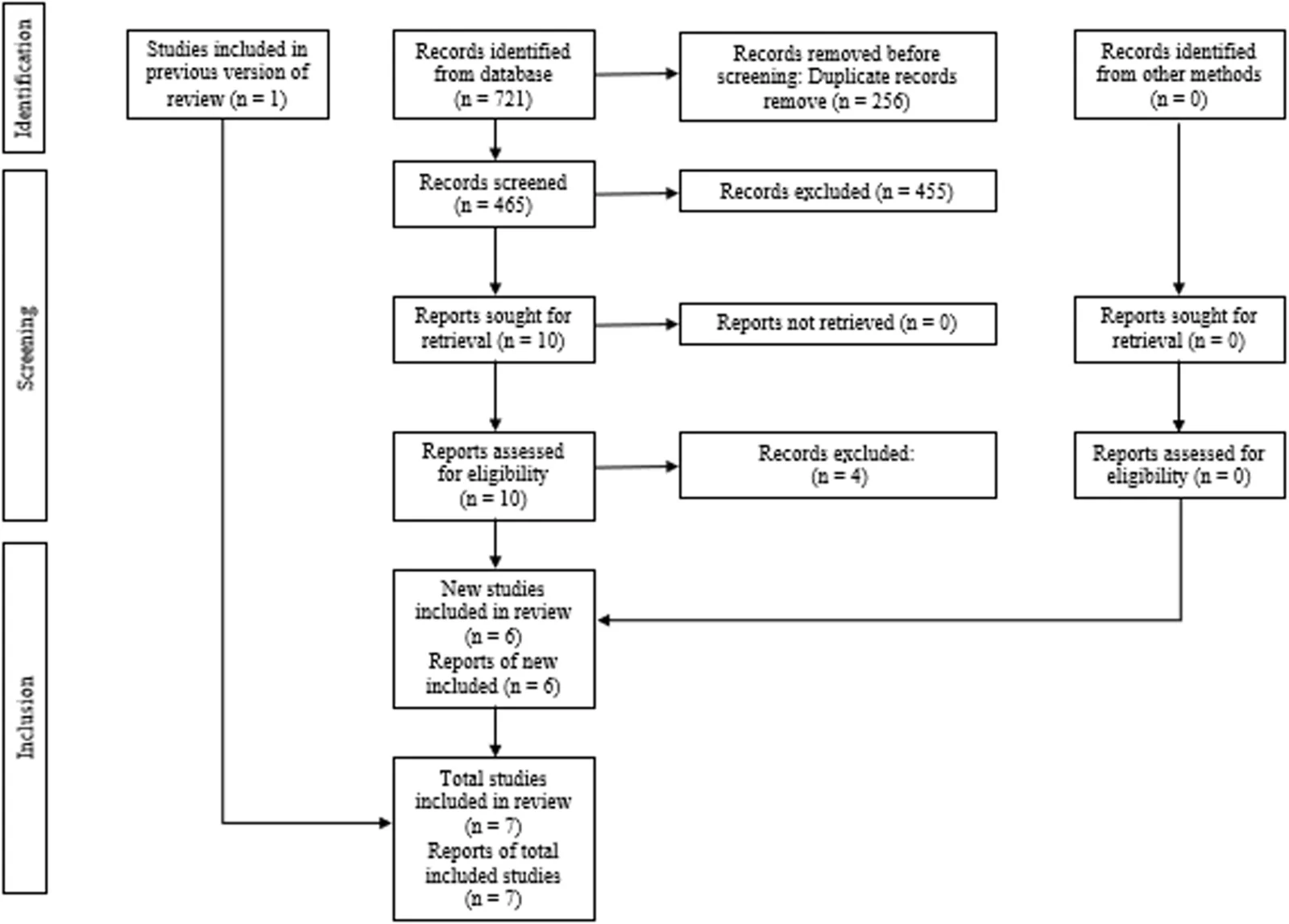
PRISMA 2020 flow diagram illustrating the study selection process. The diagram summarizes the identification, screening, eligibility assessment, and inclusion of studies according to the PRISMA 2020 guidelines. Reasons for exclusion of full-text articles are presented to ensure transparency in the selection process.

A total of 721 articles were identified through the comprehensive search. Following the removal of 256 duplicates, title and abstract screening of the 465 remaining records resulted in the exclusion of 455 studies. The 10 remaining articles underwent full-text assessment for eligibility, of which 4 were excluded (reasons summarized in Table 2). To the 6 remaining studies, 1 additional eligible study identified from a previous version of the review was added. In total, seven reports corresponding to six independent randomized clinical trials were included in the qualitative synthesis. One randomized clinical trial generated two complementary publications reporting different outcomes. Six randomized clinical trials provided data for the quantitative synthesis (meta-analysis). The overall selection process is depicted in Figure 1.

**Table 2:** Reason for exclusion of studies.

Author	Reason of exclusion
Patel et al. (37)	Conducted exclusively in permanent teeth; outside the predefined population (primary dentition).
Sharawat et al. (38)	Did not include a conventional ART comparison group; therefore, it did not meet the predefined intervention/comparator criteria.
Bansal et al. (39)	
Jiang et al. (40)	Secondary publication from the same randomized clinical trial reporting different outcomes (quality of life and parental satisfaction) rather than clinical success; excluded from quantitative synthesis to avoid duplicate participant inclusion.

### 4.2 Characteristics of included studies

#### 4.2.1 Study design and setting

A total of seven reports corresponding to six independent randomized clinical trials (41–47) published between 2020 and 2026 were included. Notably, Jiang et al. and Jiang et al. represent two complementary reports derived from the same randomized clinical trial cohort. The former reported clinical success outcomes, whereas the latter focused on parental satisfaction and oral health-related quality of life. Accordingly, only Jiang et al. contributed data to the quantitative synthesis of clinical success, thereby avoiding duplicate inclusion of participants in the meta-analysis. Four of these studies (42, 43, 46, 47) utilized a parallel-group RCT design, while the remaining three (41, 44, 45) were categorized as cross-over or split-mouth RCTs. The geographical distribution of the literature showed a concentration in developing or middle-income nations: the majority of the trials were conducted in Egypt (42–45), followed by China (46, 47), and Lebanon (41). Regarding the funding sources of the included studies, six studies reported (41–43, 45–47) their funding status (one receiving financial support and the other five receiving no financial support), whereas one trial (44) did not declare its source of funding.

#### 4.2.2 Population characteristics

Across the six independent randomized clinical trials, a total of 398 unique pediatric participants were enrolled. Because Jiang et al. (46) and Jiang et al. (47) report different outcomes from the same trial cohort, participant numbers from this study were counted only once when summarizing the study population. The participants’ ages ranged from 3 to 9 years old, adequately representing preschool and early school-aged children. The inclusion criteria across the studies were remarkably consistent; trials selected healthy children (e.g., ASA I) presenting with at least one active, cavitated carious lesion in primary molars (predominantly classified as ICDAS 4–6). Conversely, teeth exhibiting clinical or radiographic signs of irreversible pulpitis, pulpal exposure, or periapical pathology were strictly excluded from the interventions (Table 3).

**Table 3:** Characteristics of included studies.

Authors	Year	Study design	Country	Number of patients (male/female)	Average age (range)	Follow–up	Source of funding	Inclusion criteria	Exclusion criteria	Instrument - Outcome
Solh et al. (41)	2026	RCT cross-over	Lebanon	32 (18/14)	5.35 ± 0.95 (4–7)	6 months	Yes	Healthy children (ASA I), Frankl behavior 2 or 3, at least two bilateral deciduous molars with active caries (ICDAS II 4 or 5).	Systemic diseases, Frankl behavior 1 or 4, molars with previous restorations, pulp pathology or extensive structural loss (ICDAS 6).	Modified ART restoration criteria - clinical success Visual inspection and tactile examination - arrested caries A-ECOHIS - quality of life
Abdelhamid et al. (42)	2025	RCT parallel	Egypt	50 (28/22)	5.39 (4–6)	1 year	Yes	Healthy children (4–6 years) with occlusal caries (ICDAS 5 and 6) in lower second molars; cooperative; without signs of pulp involvement.	Radiographic abnormalities; symptoms of pulpitis; previous allergic reactions to materials.	ART restoration criteria - clinical success Pixel grey value - radiographic success A-ECOHIS - quality of life Directly asking - parental esthetic perception Pain reported - pain
Aly et al. (43)	2023	RCT parallel	Egypt	67 (36/31)	6.01 ± 1.15 (5–9)	1 year	Yes	Healthy children (5–9 years), positive/definitely positive behavior (Frankl 3 or 4), presence of at least one primary molar with active dentin caries on the occlusal surface.	Special health needs, silver allergy, molars with previous restorations, or signs of pulpal/periradicular pathosis.	Modified USPHS criteria (secondary caries) - clinical succes
Rady et al. (44)	2023	RCT cross-over	Egypt	25 (16/9)	4.9 ± 0.9 (4–7)	2 years	No	General health, 4–7 years, ≥ 2 deciduous molars with active dentin caries (ICDAS II 4 or 5), negative behavior (Frankl scale 2), without pulp exposure or visible periapical pathology	Silver allergy (confirmed by history and skin test), ulcerative gingivitis	Probe and percussion - clinical success Pain reported - pain
Mohammed et al. (45)	2022	RCT cross-over	Egypt	30	(3–6)	1 year	Yes	Healthy children aged 3–6 years with lower primary first molars with bilateral cavitated caries, asymptomatic or with reversible pulpitis.	Chronic systemic conditions or irreversible pulpitis.	Visual inspection and tactile examination - clinical success Catch with probe - recurrent caries Pain reported - pain
Jiang et al. (46)	2022	RCT parallel	China	194 (116/78)	(3–4)	2 years	Yes	Healthy children with at least one caries lesion in cavitated dentin, parental consent.	Severe systemic diseases, lack of cooperation, signs of pulp pathology (abscesses) or cavities too small for hand instruments.	ECOHIS - quality of life
Jiang et al. (47)	2020	RCT parallel	China	194 (116/78)	(3–4)	2 years	Yes	Healthy children with at least one caries lesion in cavitated dentin, parental consent.	Severe systemic diseases, lack of cooperation, signs of pulp pathology (abscesses) or cavities too small for hand instruments.	ART restoration criteria - clinical success

Authors	Groups	Number of patients per group	Number of teeth per group	Clinical success	Radiographic success	Arrested caries	Class I	Class II	Pain	Quality of life	Aesthetic perception	Total cost
Solh et al. (41)	SMART 38%	32	34	6m: 24/34	NR	6m: 34/34	6m: 8/10	6m: 16/24	NR	6m: 10.13 ± 6.13	NR	NR
	ART	32	34	6m: 23/34	NR	6m: 31/34	6m: 10/13	6m: 13/21	NR		NR	NR
Abdelhamid et al. (42)	SMART 38%	25	25	3m: 25/25 6m: 25/25 1y: 23/25	6m: 1.16 ± 0.07 1y: 1.21 ± 0.09	NR	NR	NR	3m: 0/25 6m: 0/25 1y: 1/25	3m: 10.40 ± 5.45	3m: 19/25	NR
	ART	25	25	3m: 25/25 6m: 24/25 1y: 22/25	6m: 1.19 ± 0.09 1y: 1.23 ± 0.11	NR	NR	NR	3m: 0/25 6m: 0/25 1y: 1/25	3m: 10.32 ± 5.45	3m: 25/25	NR
Aly et al. (43)	SMART 38%	34	59	6m: 49/51 1y: 48/50	NR	NR	6m: 49/51 1y: 48/50	NR	NR	NR	NR	60.5 ± 3.5 EP
	ART	33	60	6m: 49/52 1y: 46/49	NR	NR	6m: 49/52 1y: 46/49	NR	NR	NR	NR	67.4 ± 4.1 EP
Rady et al. (44)	SMART 38%	25	30	3m: 26/27 6m: 24/26 9m: 22/26 1y: 22/26 2y: 22/25	NR	NR	NR	NR	3m: 1/27 6m: 2/26 9m: 4/26 1y: 4/26 2y: 3/25	NR	NR	NR
	ART	25	30	3m: 23/27 6m: 17/26 9m: 16/26 1y: 16/26 2y: 15/25	NR	NR	NR	NR	3m: 7/27 6m: 9/26 9m: 10/26 1y: 10/26 2y: 10/25	NR	NR	NR
Mohammed et al. (45)	SMART 38%	30	30	6m: 23/30 1y: 18/29	NR	NR	NR	NR	NR	NR	NR	NR
	ART	30	30	6m: 21/30 1y: 16/29	NR	NR	NR	NR	NR	NR	NR	NR
Jiang et al. (46)	SMART 38%	101	260	NR	NR	NR	NR	NR	NR	6m: 7 ± 6.9 2y: 7.7 ± 7.2	NR	NR
	ART	93	249	NR	NR	NR	NR	NR	NR	6m: 7.3 ± 8 2y: 8.5 ± 6.9	NR	NR
Jiang et al. (47)	SMART 38%	101	260	6m: 150/260 1y: 120/260 1.5y: 97/260 2y: 60/208	NR	NR	NSR	NSR	NR	NR	NR	NR
	ART	93	249	6m: 150/249 1y: 121/249 1.5y: 84/249 2y: 57/212	NR	NR	NSR	NSR	NR	NR	NR	NR

RCT, randomized clinical trial; ASA, American Society of Anesthesiologists; ICDAS, International Caries Detection and Assessment System; ART, atraumatic restorative treatment; A-ECOHIS, Arabic version of the early childhood oral health impact scale; USPHS, United States Public Health Services.

SMART, silver modified atraumatic restorative treatment; ART, atraumatic restorative treatment; EP, Egyptian pounds; NR, not reported; NSR, not separately reported (the variable was considered in the study, but outcome-specific data were not presented in an extractable format); m, months; y, years.

#### 4.2.3 Interventions and follow-up

A cumulative total of 1,375 primary teeth were treated and evaluated throughout the included studies. In all trials, the experimental group was treated with SMART using 38% SDF, whereas the control group was treated with conventional ART. The follow-up durations varied depending on the study design: one study (41) evaluated outcomes at 6 months, three studies (42, 43, 45) conducted evaluations at a 1-year follow-up, and the remaining three studies (44, 46, 47) extended their observation period up to 2 years (Table 3).

#### 4.2.4 Outcome measures

The included trials evaluated a comprehensive set of both clinical and patient-centered outcomes. The primary clinical parameters measured were clinical success, radiographic success, and caries arrest rates (stratified by Class I and Class II cavities in some studies). Furthermore, several studies incorporated subjective and behavioral variables into their methodology, evaluating patient-reported pain, impacts on quality of life, parental aesthetic perception, and the overall cost of the restorative procedures (Table 3).

### 4.3 Risk of bias analysis of studies

**Figure 2:**
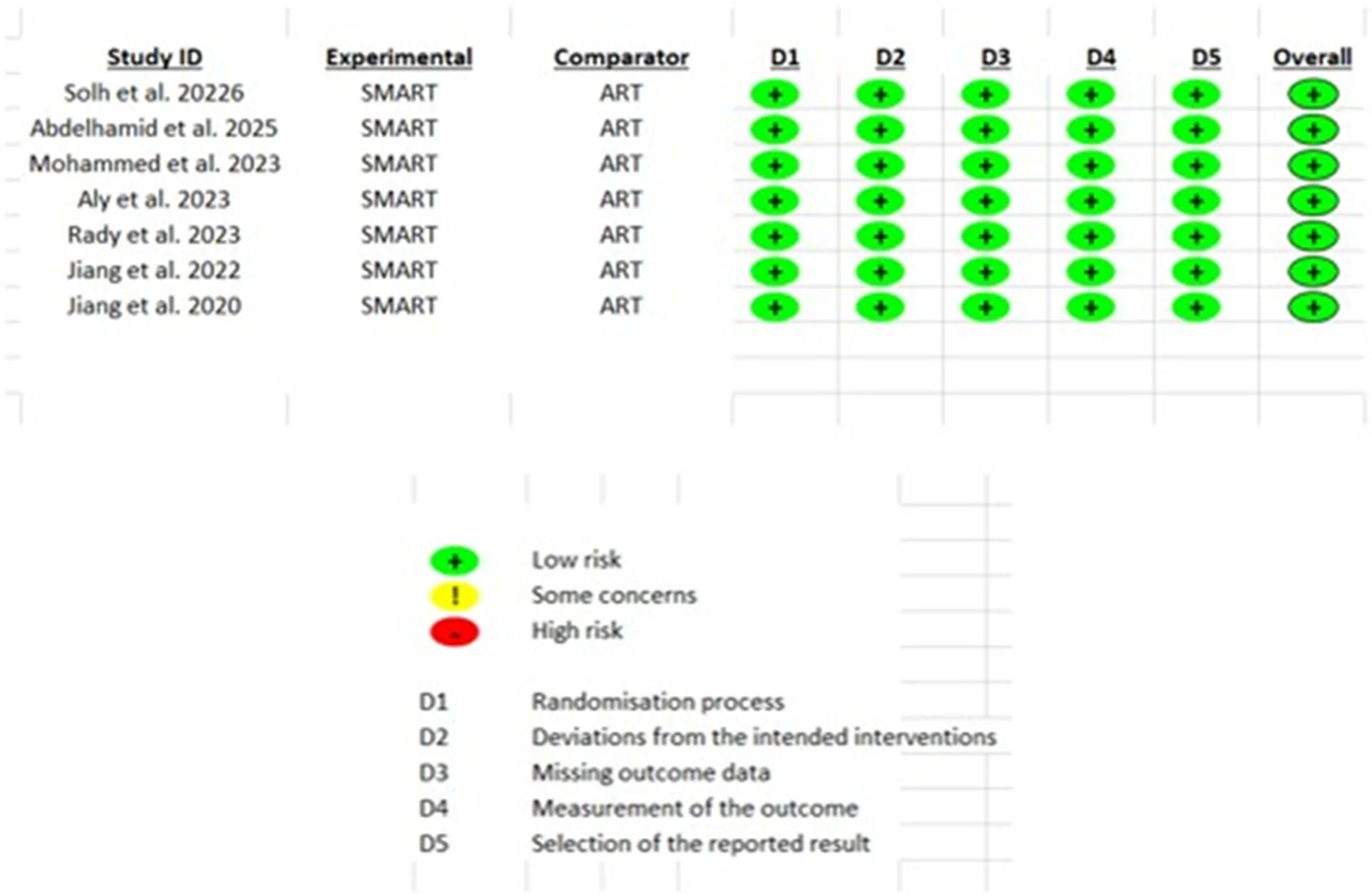
Risk-of-bias assessment using the revised Cochrane Risk of Bias tool (RoB 2). Overall judgments for each included randomized clinical trial across the five RoB 2 domains are presented. All included studies were judged to have an overall low risk of bias.

According to the RoB 2 tool, all included studies were judged to have an overall low risk of bias (Figure 2).

### 4.4 Synthesis of results (meta-analysis)

**Figure 3:**
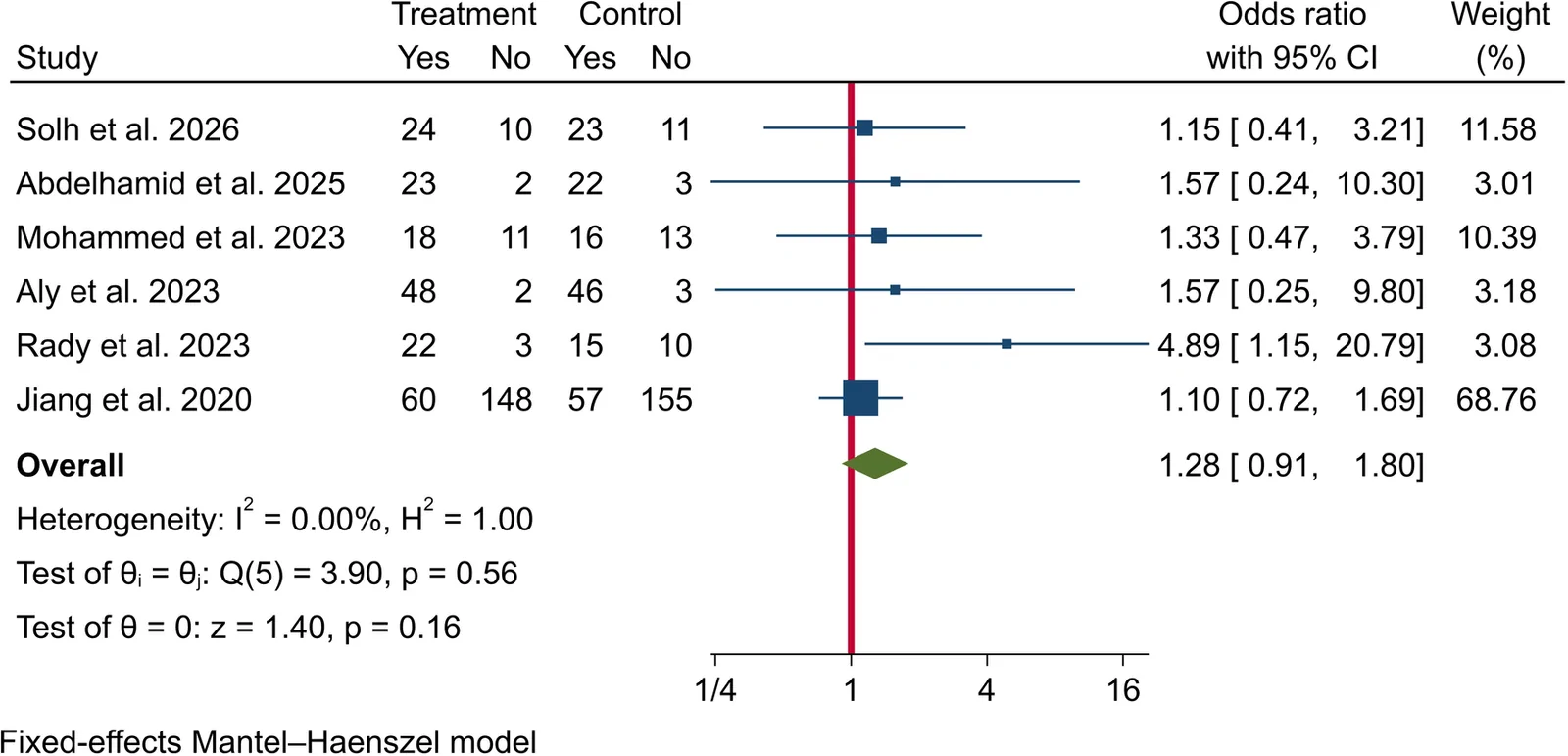
Forest plot of the meta-analysis evaluating overall clinical success of SMART versus conventional ART. The pooled odds ratio (OR) and corresponding 95% confidence interval (CI) were calculated using a fixed-effects Mantel-Haenszel model. Squares represent study-specific effect estimates, with their size proportional to study weight, while horizontal lines indicate 95% confidence intervals. The diamond represents the pooled effect estimate.

Among the predefined outcomes, only clinical success was reported with sufficient consistency across the included randomized clinical trials to allow quantitative synthesis. The remaining outcomes, including radiographic success, quality of life, pain, aesthetic perception, caries arrest, and treatment cost, were reported by a limited number of studies using heterogeneous outcome measures, follow-up periods, and assessment methods. Consequently, these outcomes were synthesized qualitatively. The meta-analysis to evaluate overall clinical success (Figure 3) included 6 RCTs (41–45, 47). A Mantel-Haenszel fixed-effects model was employed, given that no statistical heterogeneity was observed among the included studies (*I*^2^ = 0.00%, *p* = 0.56). The analysis revealed an overall Odds Ratio (OR) of 1.28 (95% CI: 0.91–1.80). Although the point estimate suggests a slight trend in favor of SMART compared to conventional ART, this difference did not reach statistical significance (*z* = 1.40, *p* = 0.16). It is worth noting that the study by Jiang et al. (47) contributed the greatest statistical weight to the analysis (68.76%).

### 4.5 Sensitivity analysis

**Figure 4:**
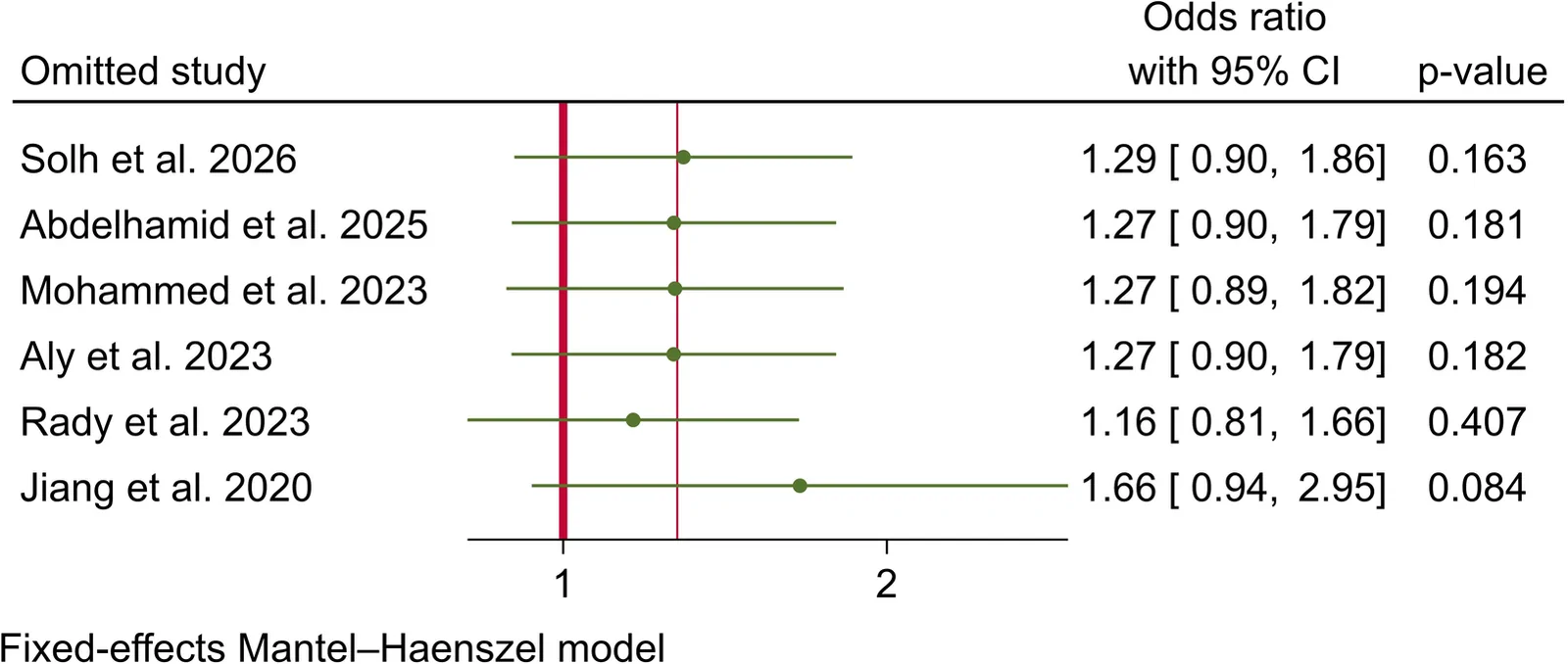
Leave-one-out sensitivity analysis. Each study was sequentially omitted from the meta-analysis to evaluate its influence on the pooled effect estimate. The stability of the overall effect demonstrates the robustness of the meta-analysis findings.

To evaluate the robustness of the findings and the individual impact of each study on the overall estimate, a sensitivity analysis was performed by sequentially omitting each study (Figure 4). The results demonstrated that the exclusion of any individual study did not significantly alter the overall result. The OR fluctuated slightly between 1.16 (when omitting Rady et al. (44)) and 1.66 (when omitting Jiang et al. (47)), but in all scenarios, the 95% confidence interval continued to cross the line of no effect (OR = 1). This confirms that the lack of a statistically significant difference between SMART and ART is a consistent finding and does not depend disproportionately on a single trial.

### 4.6 Publication bias analysis

#### 4.6.1 Visual inspection (standard funnel plot)

**Figure 5:**
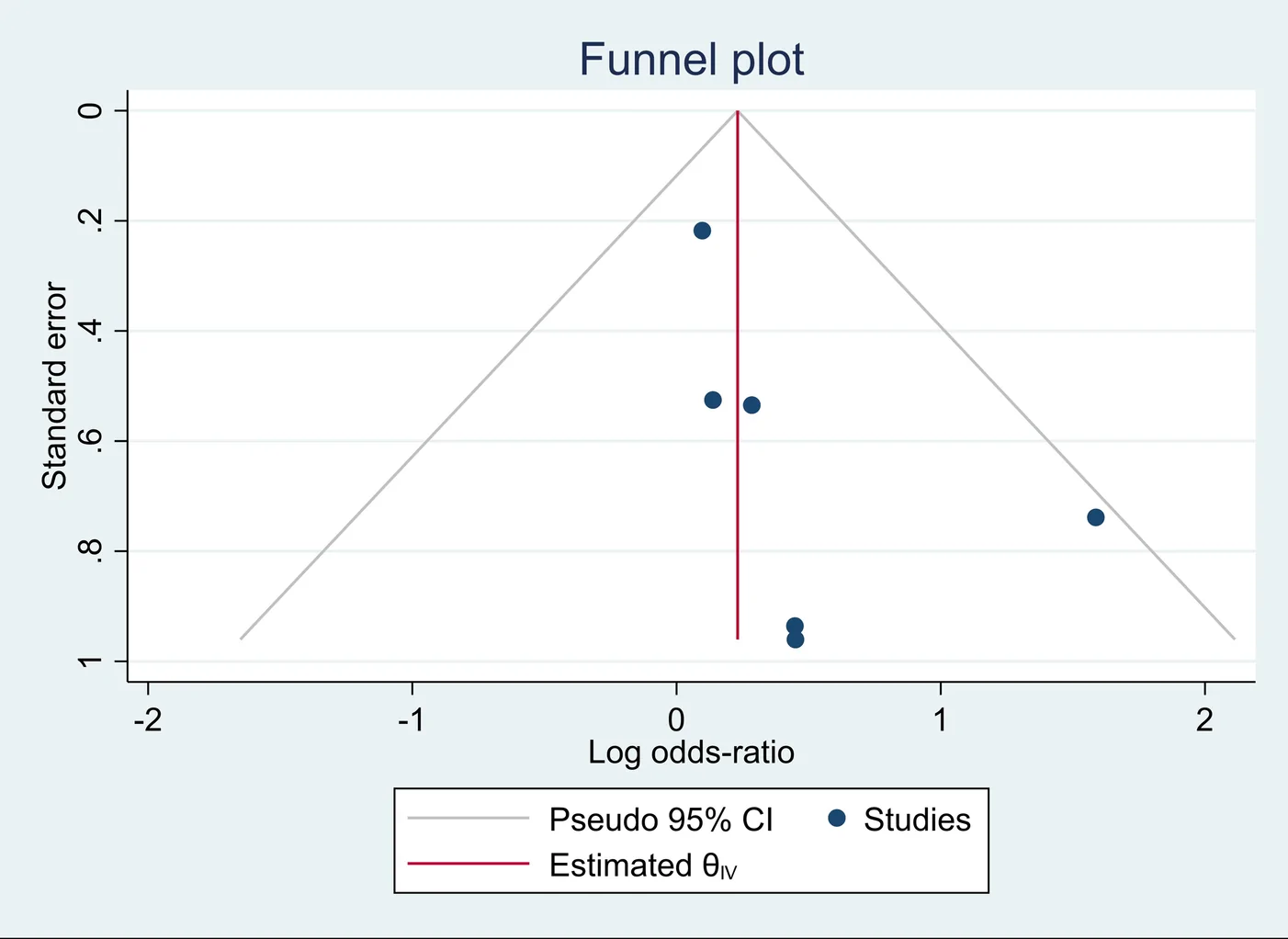
Funnel plot for visual assessment of publication bias. Each point represents an individual study plotted according to its effect estimate and standard error. Symmetry of the distribution suggests a lower likelihood of publication bias, whereas asymmetry may indicate potential small-study effects or selective publication.

The risk of publication bias was initially assessed through visual inspection of a standard funnel plot (Figure 5). The funnel plot displays a certain degree of asymmetry relative to the pooled effect estimate. Specifically, fewer studies were observed in the lower-left region of the plot, which may suggest the presence of small-study effects or potential publication bias. However, interpretation should be made cautiously because fewer than ten studies were included (k = 6), limiting the reliability of funnel plot assessments. Therefore, formal statistical tests for publication bias (e.g., Peters’ or Harbord's tests) were not performed, in accordance with Cochrane recommendations.

#### 4.6.2 Duval and Tweedie's trim and fill method

**Figure 6:**
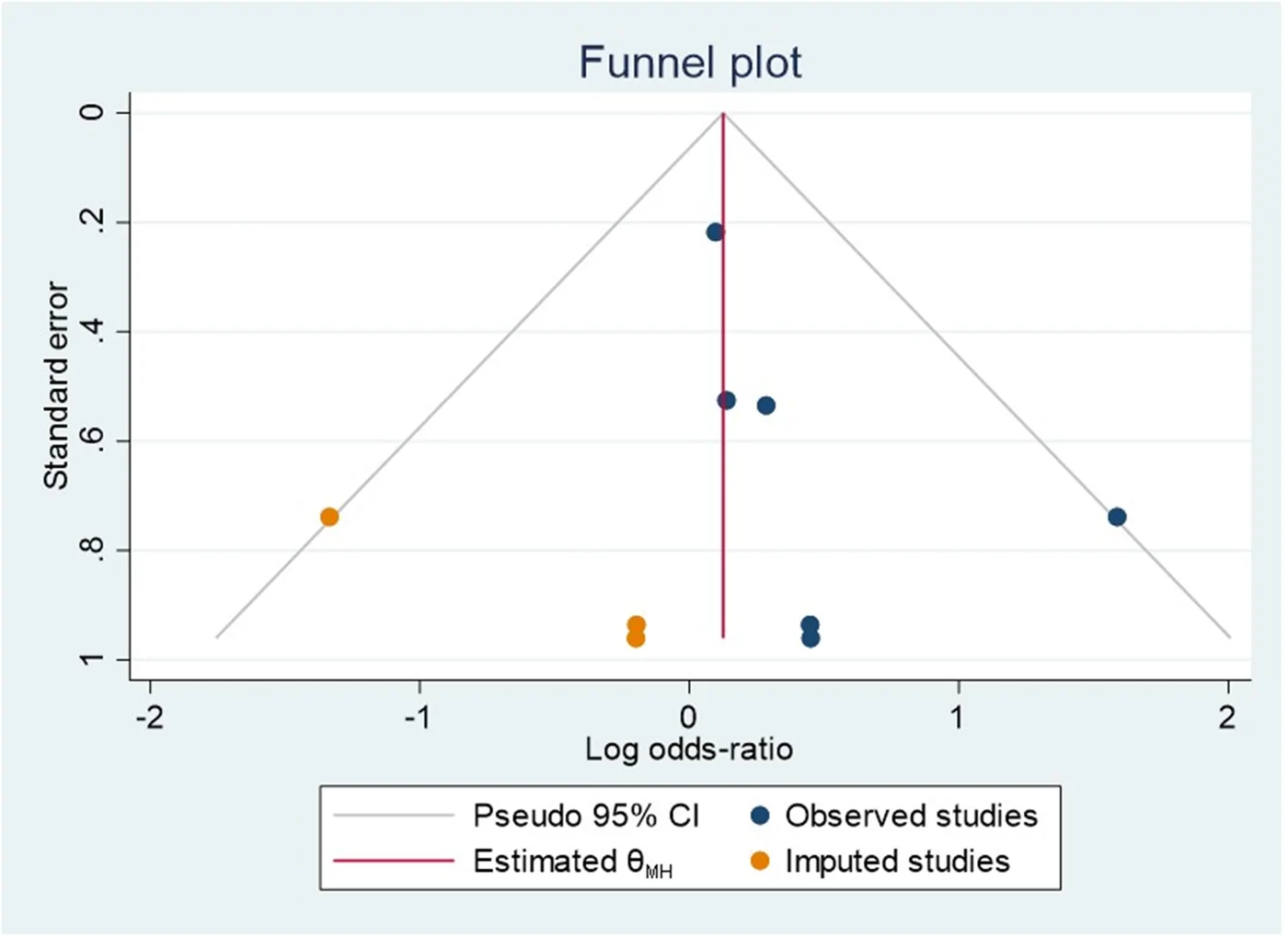
Duval and Tweedie's Trim-and-Fill analysis. The Trim-and-Fill method was applied to explore the potential influence of publication bias by estimating hypothetical missing studies and recalculating the pooled effect estimate. Filled symbols represent observed studies, whereas open symbols indicate imputed studies.

To further explore the potential impact of funnel plot asymmetry, Duval and Tweedie's non-parametric Trim-and-Fill method was applied (Figure 6). Three hypothetical studies were imputed to restore funnel plot symmetry. After adjustment, the pooled effect estimate remained non-significant, indicating that the main conclusion was not substantially altered. These findings suggest that although small-study effects or publication bias cannot be completely ruled out, they are unlikely to materially influence the overall interpretation of the results.

### 4.7 Trial sequential analysis

**Figure 7:**
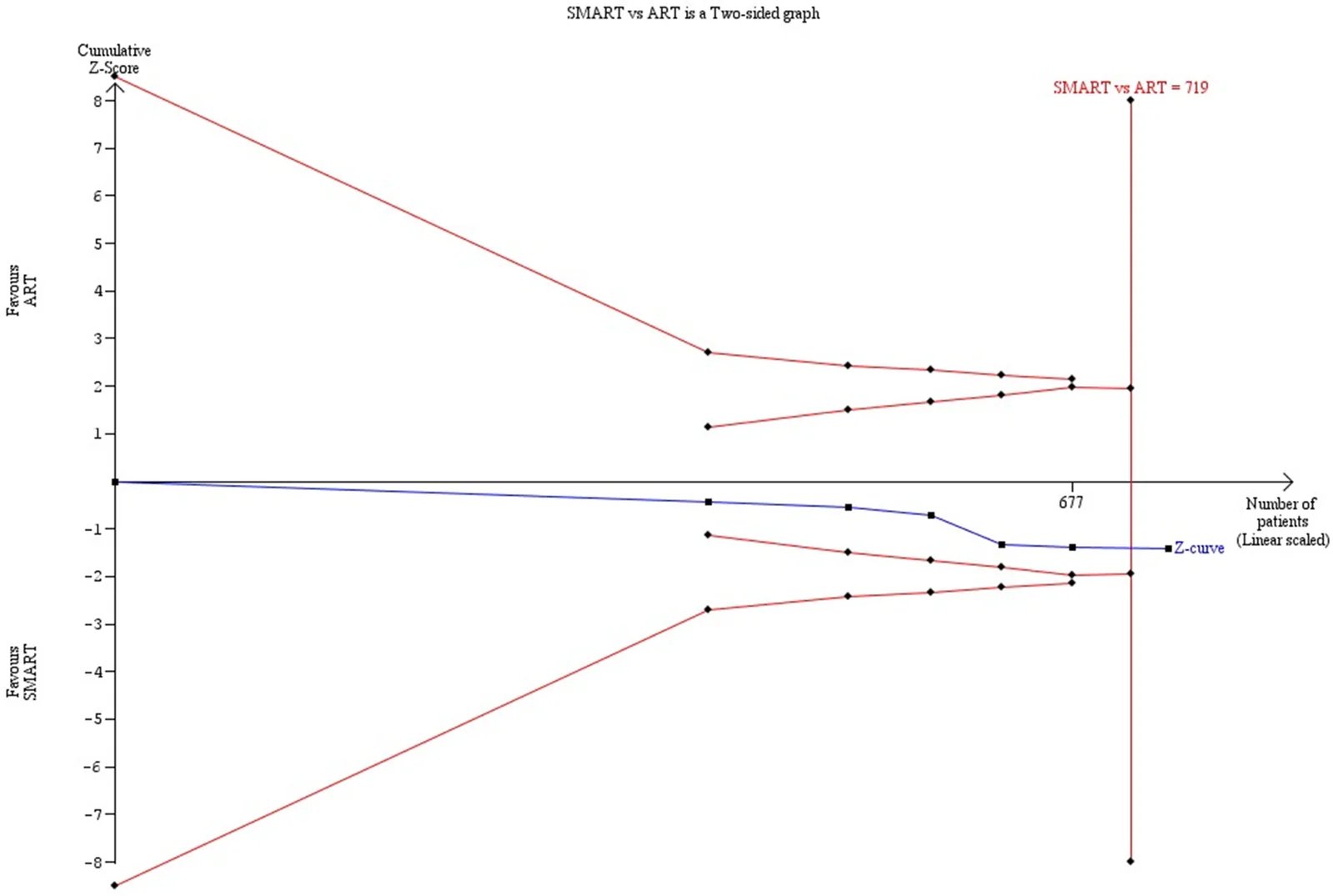
Trial sequential analysis (TSA) evaluating the sufficiency of the cumulative evidence. The cumulative Z-curve was compared with the conventional significance boundary, trial sequential monitoring boundaries, futility boundaries, and the required information size (RIS). The analysis indicates that the available evidence remains insufficient to draw definitive conclusions regarding the superiority of SMART over conventional ART.

A Trial Sequential Analysis (TSA) was conducted to determine whether the cumulative evidence is sufficient to establish firm conclusions and control the risk of random errors (Figure 7). The cumulative Z-curve (blue line) remained consistently within the futility area and did not cross the sequential monitoring boundaries (red lines with markers). Furthermore, the curve did not reach the Required Information Size (RIS), estimated at 719 patients, remaining at the mark of 677 cumulative patients. This trajectory indicates that the current evidence is inconclusive; there are insufficient data to definitively confirm or reject the superiority or inferiority of SMART compared to ART. Consequently, additional and rigorous clinical trials are required to reach the necessary sample size and provide a conclusive answer. It is crucial to highlight that this analysis is methodologically robust, as the included primary studies addressed the potential clustering effect—derived from evaluating multiple teeth per child—by utilizing split-mouth designs or statistical models such as multilevel regression and generalized estimating equations (GEE).

### 4.8 GRADE analysis

The GRADE assessment determined that the certainty of evidence for the clinical success of SMART compared to ART is low. Although the data are derived from randomized clinical trials (which initially grants a high certainty rating), the overall certainty was downgraded by two levels. This was due, firstly, to serious imprecision in the effect estimate, as the confidence interval is wide and crosses the line of no effect (OR = 0.91–1.80), generating statistical uncertainty; and secondly, to a strong suspicion of publication bias, supported by the asymmetry in the funnel plots suggesting the absence of studies with negative results. A detailed summary of the GRADE certainty assessment is presented in Table 4.

**Table 4:** GRADE analysis of included studies.

Certainty assessment							№ of patients		Effect		Certainty
№ of studies	Study design	Risk of bias	Inconsistency	Indirectness	Imprecision	Other considerations	SMART	ART	Relative (95% CI)	Absolute (95% CI)	
Clinical success (follow-up: range 6 months to 2 years)											
6	randomised trials	not serious	not serious	not serious	serious	publication bias strongly suspected	195/371 (52.6%)	179/374 (47.9%)	OR 1.28 (0.91 to 1.80)	62 more per 1,000 (from 23 fewer to 144 more)	⊕⊕◯◯ Low

CI, confidence interval; MD, mean difference; OR, odds ratio; SMART, silver modified atraumatic restorative treatment; ART, atraumatic restorative treatment.

In clinical terms, this low certainty indicates that our confidence in the current effect estimate is limited. Although the data suggest a slight mathematical advantage in favor of SMART (62 additional successes per 1,000 patients), it is highly likely that future high-quality studies will significantly modify this estimate. Consequently, the current findings should be interpreted with caution until larger and more conclusive clinical trials become available in the scientific literature.

## 5 Discussion

The primary objective of this meta-analysis was to evaluate and compare the clinical efficacy of the SMART versus conventional ART for caries control in the primary dentition. Our findings demonstrate that, although there is a slight mathematical trend in favor of SMART (OR = 1.28), there is no evidence of statistical or clinically significant superiority over conventional ART. From a clinical and practical perspective, these results suggest that both approaches are minimally invasive, viable, and equally effective therapeutic options. On a day-to-day basis, this grants the pediatric dentist the flexibility to choose either alternative based on patient behavior and treatment tolerance, knowing that the overall prognosis of both restorations remains within statistically analogous and reliable ranges, without the need for aggressive interventions.

Our results closely align with the consensus of recent systematic reviews published in the literature. Mohapatra and Mohandas (48) reported comparable clinical performance between SMART and conventional methods or ART in deciduous teeth, highlighting the absence of statistically significant differences in overall success. Along the same lines, Siang Lin et al. (49) confirmed through a two-arm meta-analysis that, although the SMART technique presented slightly higher success rates at 6 and 12 months, this point difference did not reach statistical significance. Furthermore, in a comprehensive systematic review supported by the American Dental Association, Urquhart et al. (50) reported that non-restorative interventions with SDF and sealing approaches using glass ionomer cement (GIC) exhibit high efficacies. In retrospect, contemporary evidence consolidates the premise that the addition of SDF prior to the ionomer does not confer an overwhelming superiority over the hermetic mechanical seal and nutrient deprivation already provided by the ART technique *per se*.

The complete absence of statistical heterogeneity in our overall clinical success analysis (*I*^2^ = 0.00%) is particularly noteworthy, indicating a remarkable consistency in the direction and magnitude of the treatment effects across the included studies. However, this statistical consistency should not be interpreted as the absence of clinical heterogeneity. The included randomized clinical trials differed in several clinically relevant aspects, including follow-up duration (6–24 months), cavity characteristics (Class I and Class II lesions), lesion assessment criteria, and outcome evaluation methods. Although the definition of clinical success varied among studies, the evaluated outcomes consistently reflected the overall clinical performance of the restorations and were therefore considered sufficiently comparable for quantitative synthesis. Nevertheless, despite these clinical differences, the pooled estimates remained highly consistent, suggesting that the comparative effectiveness of SMART and ART was robust across diverse clinical settings. This finding contrasts with previous evidence from systematic reviews focusing exclusively on silver diamine fluoride, where greater statistical heterogeneity has been reported, likely reflecting the broader variability in intervention protocols and outcome assessment across studies (51). As well documented by Siang Lin et al. (49), the marked divergences in longitudinal success rates can be attributed to the disparity in evaluation criteria; studies focusing on “marginal adaptation” reported significantly higher rates (around 91.1% at 6 months), contrasting sharply with those that measured failure through subjective or symptomatic variables such as “pathological mobility” or “pain.” Likewise, the decline in the success rate over time—dropping from 94.9% at 3 months to 69.5% by 12 months—appears inherent to the restorative material and depends on factors such as cavity size (Class I vs. Class II), isolation of the operative field, and follow-up duration (which in our cohort ranged from 6 to 24 months).

Radiographic outcomes were evaluated in only one randomized clinical trial included in this review (42), which demonstrated comparable radiographic findings between the SMART and ART groups throughout the follow-up period. Although quantitative synthesis was not feasible because only a single study assessed this outcome, the available evidence suggests that prior application of SDF does not adversely influence the radiographic performance of subsequent ART restorations. Although based on a single randomized clinical trial, these findings are biologically plausible because SDF promotes caries arrest through its antimicrobial activity and remineralization potential (19, 27, 52), while previous evidence has shown that it does not compromise the bonding performance of glass ionomer cements used in ART restorations (26). However, the current radiographic evidence remains limited, and additional randomized clinical trials incorporating standardized radiographic outcome measures are required before definitive conclusions regarding long-term radiographic success can be established.

Although the meta-analysis demonstrated remarkable consistency for the primary outcome of clinical success, substantially greater heterogeneity was observed among the predefined secondary outcomes. This heterogeneity was not only quantitative but also methodological, arising from differences in outcome definitions, assessment instruments, follow-up schedules, and reporting practices across the included trials. Quality of life, for example, was evaluated using the A-ECOHIS questionnaire in only two studies, whereas radiographic success, parental aesthetic perception, and treatment costs were each evaluated in a single trial. Likewise, pain and caries arrest were reported using different methodologies and at different follow-up intervals. Consequently, these outcomes could not be quantitatively synthesized, limiting direct comparisons and preventing robust conclusions regarding their relative benefits beyond clinical success. Nevertheless, the available evidence suggests that these patient-centered outcomes represent clinically relevant domains that deserve greater methodological consistency in future randomized clinical trials.

From a theoretical and biological rationale, it is plausible to theorize why the long-term performance of both techniques ultimately balances out, despite the slight mathematical trend of 62 additional successes per 1,000 patients in favor of SMART. SMART's initial boost lies in the synergistic effect of its materials, highlighting the dual mechanism of 38% SDF: silver ions exhibit profound antimicrobial action by destroying cell membranes and blocking metalloproteinases, while the high fluoride content interacts to form acid-resistant fluorhydroxyapatite (27, 52). Subsequently, the GIC establishes a double barrier by sealing the cavity and biologically asphyxiating the remaining biofilm. However, as argued by Siang Lin et al. (49), this theoretical biochemical advantage conferred by the SDF is diluted against the purely mechanical efficacy of ART in the medium and long term. This responds to the physical wear and chemical degradation to which the GIC is prone under masticatory stress, a material fatigue that eventually leads to marginal microleakage or secondary caries.

The interpretation of these findings must be made while recognizing certain fundamental limitations. Although all included randomized clinical trials evaluated the overall clinical success of minimally invasive restorative treatment, the definition of clinical success was not fully standardized across studies. Different assessment criteria were used, including visual and tactile examination, modified USPHS criteria, pain, marginal adaptation, and other clinical indicators. Although these measures reflected comparable constructs of restorative performance and were therefore considered suitable for quantitative synthesis, this methodological variability should be taken into account when interpreting the pooled estimates. Furthermore, several predefined secondary outcomes, including radiographic success, quality of life, pain, parental aesthetic perception, and treatment cost, were reported inconsistently and using heterogeneous outcome definitions, assessment instruments, and follow-up schedules. Consequently, these outcomes could only be synthesized qualitatively, preventing robust quantitative comparisons and limiting the comprehensiveness of the available evidence. According to the GRADE system, the certainty of the current evidence is “low,” penalized primarily by serious imprecision, where wide confidence intervals cross the line of no effect, and by asymmetry in the funnel plot, warning of a strong suspicion of publication bias due to a lack of literature reporting negative results. Additionally, the analysis is hindered by intrinsic limitations of the primary studies, such as the risk of attrition bias and the lack of standardization in success rubrics. Despite this, our research has important methodological strengths: the control of clustering bias, the robustness of the sensitivity analysis, and the application of the Trim and Fill method, which proved that the “missing” studies do not invert our central conclusion. Of equal value, the rigorous TSA empirically demonstrated that the Z-curve remains in the futility zone, mathematically validating that with 677 documented patients, the RIS threshold of 719 patients has not yet been crossed to dictate an unappealable conclusion. At the review level, no individual participant data were available, subgroup analyzes could not be performed because of the limited number of studies, and publication bias could only be explored through visual methods and Trim-and-Fill because fewer than ten studies were available.

In the real world of pediatric dentistry and public health, assimilating that the SMART technique and ART are statistically comparable empowers clinicians to make more pragmatic decisions. Given that the application of SMART requires considerably shorter working times (approximately half that of conventional ART) and is highly cost-effective, it positions itself as an exceptional strategic intervention for pediatric populations with extreme anxiety, behavior management problems, or in community programs. It allows adapting the treatment plan based on these determinants, knowing that both pathways (SMART and ART) are safe. In conclusion, current evidence demonstrates that both therapeutic strategies are minimally invasive and equally effective for caries control, with no evidence supporting a definitive superiority of one over the other. However, the evident gaps in accumulated statistical power make it imperative to design multicenter and standardized clinical trials that surpass the sample volume dictated by the TSA to issue definitive recommendations on their long-term behavior. Future studies should also adopt standardized definitions of clinical and patient-centered outcomes, validated assessment instruments, consistent follow-up periods, and transparent reporting of radiographic success, quality of life, pain, parental aesthetic perception, and treatment costs to facilitate meaningful comparisons and future quantitative evidence synthesis.

## 6 Conclusions

In conclusion, current evidence suggests that there is no statistically significant difference in overall clinical success between SMART and conventional ART for caries control in the primary dentition. Although the SMART technique offers the additional biological benefit of arresting caries progression through the use of silver diamine fluoride (SDF), the certainty of the available evidence is low due to statistical imprecision and the risk of publication bias. Furthermore, the trial sequential analysis (TSA) confirms that the current data are inconclusive. Therefore, both techniques remain viable, effective, and patient-centered minimally invasive strategies. To establish definitive clinical guidelines, it is essential to conduct future large-scale randomized controlled trials that can successfully reach the required information size.

## Data Availability

The original contributions presented in the study are included in the article/Supplementary Material, further inquiries can be directed to the corresponding author.
